# Quantitative analysis of retinal microvascular changes in gestational hypertension and preeclampsia using artificial intelligence technology

**DOI:** 10.3389/fcell.2026.1873901

**Published:** 2026-07-01

**Authors:** Tingting Hu, Shen Hu, Di Luo, Yanli Jiang, Yajie Zheng, Jie Wang, Xingye Wang, Qiong Wang, Fangfang Chen

**Affiliations:** 1 Dadukou District People’s Hospital, Chongqing, China; 2 Department of Obstetrics, The Second Affiliated Hospital, Zhejiang University School of Medicine, Hangzhou, China; 3 The Harvard T.H. Chan School of Public Health, Boston, MA, United States; 4 Shapingba Chenjiaqiao Hospital, Chongqing, China; 5 EVision Technology (Beijing) Co. Ltd., Beijing, China

**Keywords:** artificial intelligence, early diagnosis, hypertensive disorders of pregnancy, preeclampsia, proteinuria, retinal microvasculature

## Abstract

Hypertensive disorders of pregnancy (HDP) are among the leading causes of maternal and perinatal morbidity and mortality worldwide. Current clinical diagnosis primarily relies on late-onset indicators including elevated blood pressure and proteinuria, which fail to meet clinical demands for early identification and risk stratification. The retinal microvasculature shares high structural and physiological homology with cerebral and placental vasculature, providing an ideal non-invasive window for systemic microcirculation assessment. In this exploratory cross - sectional study, artificial intelligence - based quantitative fundus analysis was employed to assess retinal microvascular parameters in 36 pregnant women (72 eyes). The retinal arterial diameter and the mean macular venous diameter were notably narrower in both the gestational hypertension (GH) and preeclampsia (PE) groups when compared with the healthy control group (all p < 0.001), and there was no significant difference between the two disease groups. In univariate analyses, serum uric acid exhibited a stronger negative correlation with the arterial diameter (r = −0.614) than diastolic blood pressure (r = −0.436). In multivariable generalized estimating equation (GEE) models that accounted for inter - eye correlation and maternal covariates, serum uric acid remained independently associated with a narrower retinal arterial caliber, while diastolic blood pressure was independently associated with a reduced macular venous caliber. No independent associations were detected for the arteriovenous ratio or fractal dimension. Vascular density and the arterial branching angle were significantly correlated with 24-h urinary protein excretion, and the arterial branching angle was the sole parameter showing a between - group difference between GH and PE. These findings imply that the narrowing of the retinal vascular caliber represents a consistent microvascular alteration in hypertensive disorders of pregnancy (HDP), with differential arterial - venous association patterns observed in multivariable analyses. AI - assisted quantitative retinal assessment offers an objective framework for investigating systemic microvascular alterations in HDP and requires validation in larger longitudinal studies.

## 1 Introduction

Hypertensive disorders of pregnancy (HDP) are leading causes of global maternal and perinatal morbidity and mortality, accounting for approximately 76,000 maternal deaths and 500,000 infant deaths worldwide each year (Duley, 2009; Vera-Ponce et al., 2025). HDP imposes a considerable global disease burden; a systematic review indicated that the global prevalence rates of preeclampsia (PE), eclampsia, and HELLP syndrome are 4.43%, 0.43%, and 0.39%, respectively (Abalos et al., 2013). Beyond causing adverse acute pregnancy outcomes, HDP signifies a systemic vascular dysfunction that notably heightens patients’ long-term risks for chronic hypertension, subsequent cardiovascular diseases, stroke, metabolic syndrome, cognitive impairment, and chronic end-stage renal disease later in life (Brown et al., 2018; Poon et al., 2019; Callaway et al., 2007). However, the early clinical manifestations of HDP are typically insidious and non-specific. The diagnosis is usually only established after 20 weeks of gestation when hypertension, proteinuria, or organ dysfunction becomes evident, at which point endothelial damage and microvascular abnormalities have already occurred (Chaemsaithong et al., 2022). Although risk factor screening based on the NICE/ACOG guidelines holds clinical value, its sensitivity is constrained by gestational age-specific windows, individual heterogeneity, and associated testing costs, thereby complicating large-scale early screening efforts (Acog Committee Opinion No, 2018). Currently, there remains a lack of widely accessible, non-invasive biomarkers that can dynamically reflect the microvascular pathological progression associated with placental diseases. The existing assessment tools, which include maternal clinical characteristics, blood pressure indices, and serum biomarkers, provide only limited information for effective risk stratification (Saccone et al., 2026).

Retinal microvascular alterations are closely associated with the onset and progression of hypertensive disorders of pregnancy. Hypertensive disorders of pregnancy are characterized by systemic endothelial dysfunction and microvascular remodeling (Yu et al., 2018; Rana et al., 2019). Blood pressure reflects the hemodynamic load imposed on the vascular system and directly influences arteriolar tone and structural adaptation, with retinal arteriolar narrowing serving as a marker of hypertensive microvascular injury (Tripathy and Arsiwalla, 2026). Serum uric acid is independently correlated with a reduction in vessel density within the retinal capillary plexus. This suggests that uric acid may exert an influence on retinal microcirculatory parameters through microvascular injury mechanisms (Yang et al., 2022). In addition, the 24 - hour urinary protein level, serving as a marker of systemic endothelial dysfunction, exhibits a positive correlation with the prevalence and severity of retinal microangiopathy in the preeclampsia population (Tadin et al., 2001). Abnormalities in multiple vascular parameters can be detected prior to the emergence of clinical manifestations, underscoring their critical value for early disease identification (Lupton et al., 2013; Soullane et al., 2024). Early-onset preeclampsia is attributed to impaired remodeling of spiral arteries, which leads to placental dysplasia. In contrast, late-onset preeclampsia results from the interplay of physiological placental senescence, maternal genetic susceptibility to cardiovascular diseases, and predisposition to metabolic disorders (Burton et al., 2019; Brosens et al., 2011). Both retinal and placental vessels are integral components of the terminal microcirculatory system, exhibiting highly similar pathophysiological characteristics in the regulation of angiogenesis, maintenance of endothelial function, and mechanisms of blood flow autoregulation (Rana et al., 2019; Burton and Fowden, 2015). Previous studies have identified microvascular changes analogous to hypertensive retinopathy in patients with HDP, such as retinal arteriolar narrowing, a decreased arteriovenous ratio (AVR), reduced vascular density, and morphological remodeling of the vasculature. These alterations reflect maternal systemic endothelial dysfunction and abnormal microcirculatory perfusion (Lupton et al., 2013; Ciloglu et al., 2019). However, earlier investigations predominantly employed conventional ophthalmoscopy or subjective grading systems, which lack objective quantitative standards for assessing vascular structure. This limitation hinders the reproducibility of results and their potential for clinical application. Consequently, there is an urgent need for a non-invasive, quantifiable, and widely implementable tool for assessing microvascular function, which holds significant clinical importance for the early identification and risk stratification of HDP.

In this context, artificial intelligence has experienced a rapid expansion in the diagnosis of ophthalmic diseases (Zhao et al., 2022; Cao et al., 2025). This exploratory cross-sectional study applies artificial intelligence (AI)-powered quantitative fundus imaging technology to HDP research, enabling unbiased automated quantification of retinal microvascular biomarkers from color fundus photographs, which provides a methodological foundation for subsequent investigation of potential non-invasive markers for early disease identification and progression evaluation. By comparing retinal microvascular parameter differences across gestational age-matched cohorts of healthy pregnant women, patients with gestational hypertension (GH), and patients with preeclampsia, we aimed to quantitatively characterize retinal microvascular alterations in cases of gestational hypertension and preeclampsia; and to investigate whether retinal vascular phenotypes are differentially associated with key clinical indicators such as blood pressure, serum uric acid, and 24-h urinary protein excretion. Furthermore, we will analyze correlations between quantitative fundus indices and clinical laboratory parameters to provide exploratory evidence regarding associations between retinal microvascular parameters and HDP severity.

## 2 Materials and methods

### 2.1 Study population

**FIGURE 1:**
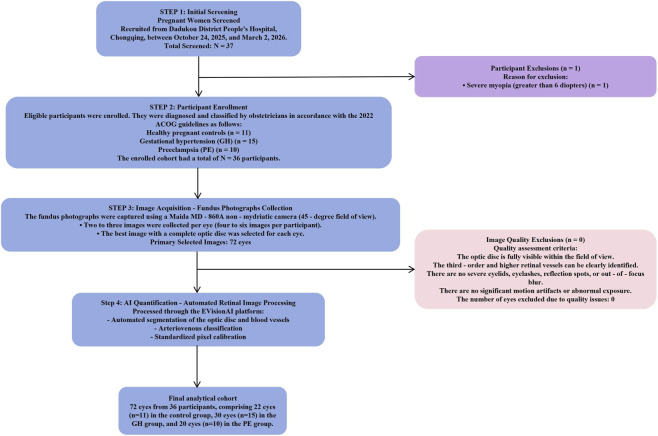
Recruitment and image-exclusion flow diagram.

Between 24 October 2025, and 2 March 2026, a total of 37 individuals were recruited from Dadukou District People’s Hospital in Chongqing, China. One participant was excluded on account of high myopia (greater than 6 diopters), leading to 36 eligible participants. Among them, there were 11 healthy pregnant women, 15 patients with gestational hypertension (GH), and 10 patients with preeclampsia (PE). Color fundus photographs were successfully acquired from all the included participants, and no images were excluded because of quality issues. The final analysis involved 72 eyes (Figure 1). Healthy pregnant women were recruited during the same period from the same hospital and frequency-matched to the HDP groups by gestational age and maternal age. Given the relative rarity of HDP (particularly PE) and strict inclusion/exclusion criteria applied, the effective sample size is somewhat limited. However, all enrolled cases underwent rigorous quality control, ensuring high completeness of clinical and imaging data, thus enhancing the reliability of the study results. All participants received systematic clinical assessments, which included baseline demographic characteristics, gestational age, obstetric history, body mass index (BMI), blood pressure measurements, past medical history, current medication status, and evaluations of pregnancy-related complications. All patients were diagnosed and classified by specialized obstetricians in accordance with the 2022 guidelines established by the American College of Obstetricians and Gynecologists (ACOG). Gestational hypertension was defined as new-onset systolic blood pressure ≥140 mmHg or diastolic blood pressure ≥90 mmHg occurring after 20 weeks of gestation on at least two occasions ≥4 h apart in previously normotensive women, without proteinuria or end-organ dysfunction. Preeclampsia was diagnosed in the presence of new-onset hypertension after 20 weeks accompanied by either proteinuria (≥300 mg/24 h, protein/creatinine ratio ≥0.3, or dipstick ≥2+) or, in the absence of proteinuria, any severe feature including thrombocytopenia, renal insufficiency, impaired liver function, pulmonary edema, or new-onset cerebral or visual symptoms (Author Anonymous, 2020). This study adhered to the ethical standards outlined in the 1964 Declaration of Helsinki and received approval from the Ethics Committee of Dadukou District People’s Hospital in Chongqing (Ethics Approval No.: CQDDKYY-LL-2025-KY-012). All participants provided written informed consent prior to enrollment.

### 2.2 Inclusion and exclusion criteria

Eligible participants met the following inclusion criteria: aged ≥18 years, gestational age ≥20 weeks, and confirmed HDP diagnosis in accordance with the 2022 ACOG guidelines. The included HDP subtypes comprised GH and PE. Subtype classification was based on blood pressure levels, proteinuria status, and presence of severe features. All diagnoses and subtype assignments were independently verified by two specialized obstetricians to ensure diagnostic accuracy and consistency.

Exclusion criteria included participants with comorbid diabetic retinopathy, retinal vascular occlusion, high myopia (greater than 6 diopters), severe opacities of the refractive media, or a prior history of ophthalmic surgery. Additionally, pregnant women with multiple gestations were excluded to minimize the potential impact of confounding factors on the study results.

### 2.3 Clinical data collection and quality control

Demographic and obstetric characteristics, such as maternal age, height, body weight, gestational age, gravidity, and parity, were retrieved from the hospital’s electronic medical record system. Body mass index (BMI) was computed as the weight in kilograms divided by the square of the height in meters (kg/m^2^). Blood pressure was measured using a calibrated automated sphygmomanometer with the participants in a seated position after a minimum of 5 min of rest. Two measurements were taken at an interval of at least 4 min and averaged for analysis. Venous blood samples were collected following an overnight fast for laboratory evaluation, including fasting blood glucose and serum uric acid. All biochemical analyses were conducted in the hospital’s central laboratory using standardized automated analyzers under routine internal quality - control procedures. Urine protein grade was evaluated by standard dipstick testing, and 24-h urinary protein excretion was measured through a complete 24-h urine collection under standardized clinical guidelines, with the samples analyzed in the same certified laboratory. All clinical variables were independently extracted from the electronic medical record system and cross - verified by two investigators to ensure data accuracy, and any discrepancies were resolved through a review of the original source documents.

### 2.4 Retinal image acquisition

Fundus images were captured using a Maida MD-860A non-mydriatic digital fundus camera (Maida Digital Medical Equipment Co., Ltd., China). Considering the unique physiological status of pregnant individuals, and to avoid potential adverse reactions to mydriatic agents, all imaging procedures in this study were conducted in non-mydriatic mode with a 45° field of view setting. Posterior pole fundus images centered on the optic disc were uniformly collected from all participants. Image acquisition was performed by two ophthalmic technicians who underwent standardized training. Prior to imaging, participants were instructed to remove their glasses, adjust the height of the chin rest and forehead position to ensure that both eyes were at the same horizontal level as the lens, and to gaze at the camera’s internal fixation light to ensure the optic disc was centered in the field of view. Two to three consecutive images were captured for each eye, and the image with the highest clarity and the most complete display of the optic disc was selected for subsequent analysis.

### 2.5 Image quality assessment and exclusion criteria

All collected fundus images underwent independent masked quality assessment by two ophthalmologists with >5 years of clinical experience. Assessors received uniform training on scoring criteria prior to the assessment, and the intraclass correlation coefficient (ICC) of the pre-assessment was 0.92 (95% CI: 0.87–0.94), indicating good inter-rater reliability. Included images met all of the following criteria simultaneously (Duley, 2009): The image fully covered the optic disc and all retinal vessels within 1.5 optic disc diameters (PD) around the optic disc (Vera-Ponce et al., 2025); There were no significant motion artifacts, out-of-focus blur, overexposure (brightness ≥240), or underexposure (brightness ≤30) (Abalos et al., 2013); Retinal arterioles and venules were clearly distinguishable up to the third-order branches. Images were excluded if they met any of the following criteria (Duley, 2009): The optic disc was not entirely visible within the image field of view (Vera-Ponce et al., 2025); Image clarity was insufficient to clearly identify third-order and higher retinal vessels (Abalos et al., 2013); The optic disc or surrounding vascular regions were obscured by eyelids, eyelashes, reflection spots, or other artifacts (Brown et al., 2018); Significant motion artifacts or abnormal exposure were present. Disagreements between the two assessing ophthalmologists were resolved by final arbitration from a senior ophthalmologist with a senior professional title. A total of 72 fundus images from 72 eyes were collected in this study, all of which met the inclusion criteria after quality assessment, and no images were excluded.

### 2.6 Quantitative analysis of retinal images

Compared to traditional manual grading methods, automated retinal vascular measurement offers greater objectivity and reproducibility. Recent advancements in artificial intelligence have further enabled automated structural analysis of ocular images, especially in chronic ocular diseases (Xu and Yang, 2023). Previous studies have demonstrated that the intraclass correlation coefficient (ICC) for computer-aided semi-automatic measurement methods can exceed 0.90, indicating a high level of consistency with manual measurement results (Cheung et al., 2010; Neubauer et al., 2008). In recent years, fully automated measurement methods based on deep learning have been shown to achieve diagnostic performance equivalent to that of semi-automatic software, with arteriovenous recognition accuracy exceeding 98% (Yan et al., 2024; Long et al., 2022).

In this study, standardized color fundus photography was conducted on all participants using a non-mydriatic fundus camera, with images collected centered on the optic disc. All images underwent quality screening prior to inclusion in the analysis. Low-quality retinal images, such as those that are blurred or underexposed, may compromise automated vessel segmentation performance and quantitative analysis accuracy (Wan et al., 2021). Therefore, images that did not meet predefined clarity and segmentation criteria were excluded to ensure the accuracy of parameter extraction. Quantitative analysis of retinal microvascular parameters was performed using intelligent fundus image analysis software (EVisionAI, Yiwei Technology (Beijing) Co., Ltd.) (Xu et al., 2019). This software, based on an edge extraction algorithm that integrates deep learning and a visual attention mechanism, performs image preprocessing, which includes region of interest (ROI) extraction, denoising, normalization, and enhancement. It then conducts hierarchical feature extraction and global context modeling through a multi-stage convolution encoder and stacked Transformer modules, ultimately generating accurate segmentation maps of the optic disc and retinal vessels while completing arteriovenous classification (Xu et al., 2019; He et al., 2023). The AI model (TransUnet architecture integrated into the EVisionAI platform) was developed and validated on a large-scale multi-center dataset including the Beijing Eye Study (BES) cohort, with proven favorable robustness and reliability of its measurement outputs (He et al., 2023; Shao et al., 2021).

**FIGURE 2:**
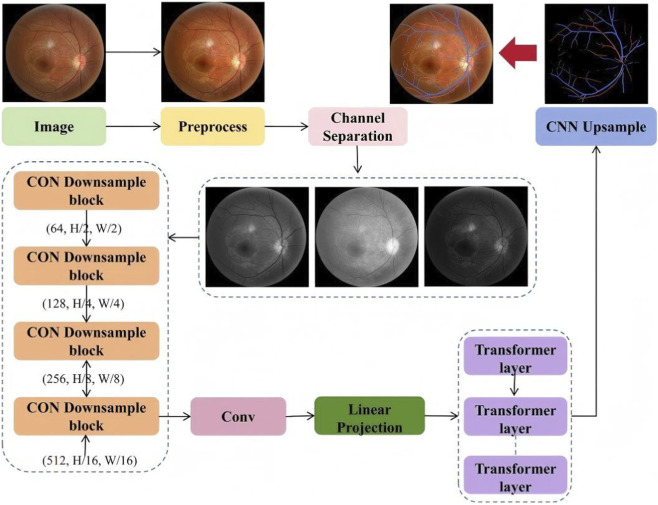
Flowchart of artificial intelligence-assisted retinal image processing and quantitative analysis.

All quantitative parameters were measured within a standardized polar coordinate framework, with the center of the optic disc serving as the origin. The definition of the measurement area was consistent with that used in previous large-scale epidemiological studies (Cheung et al., 2010; Knudtson et al., 2003). Each quantitative parameter was converted from pixel units to micrometers through a standardized pixel spacing calibration formula. Resolution was defined as the actual physical length corresponding to a single pixel (μm/pixel), calculated using the following formula: Resolution = 1921/Width _ROI_ × 6.404. This calibration method has been validated in general pixel calibration studies for fundus cameras, facilitating the comparability of quantitative parameters across images collected by different devices (Long et al., 2022). Utilizing the calibrated mapping relationship between pixels and physical units, the system can automatically generate multi-dimensional quantitative indicators that encompass retinal microvascular structure, morphology, and geometric features, including vascular fractal dimension (FD), vascular caliber (VC), vascular tortuosity (VT), vascular density (VD), vascular branching angle (VBA), arteriovenous ratio (AVR), and cup-to-disc ratio (C/D), among others (Figure 2). These quantitative fundus parameters characterize complementary aspects of the structure and function of the retinal microvasculature. The vascular diameter and arteriovenous ratio (AVR) reflect the vascular caliber, arteriolar narrowing, and systemic microvascular remodeling. The vascular density, curvature, and fractal dimension describe the richness, geometric configuration, and branching complexity of the retinal vascular network, offering insights into the perfusion status and hemodynamic stress. Optic disc–related parameters (e.g., cup-to-disc ratio and neuroretinal rim width) indicate structural remodeling and loss of neuroretinal tissue, whereas geometric measurements of the main vascular arch quantify the deformation of the posterior pole. Collectively, these metrics provide a multidimensional assessment of the retinal microcirculation and structural integrity.

### 2.7 Statistical analysis

Statistical analyses were conducted using SPSS version 27.0 (IBM Corp., Chicago, IL, United States). Continuous variables conforming to normal distribution were expressed as mean ± standard deviation (SD), while skewed distributed variables were expressed as median (interquartile range, IQR), and group comparisons were performed using the Kruskal–Wallis test, with pairwise between-group comparisons adjusted using the Bonferroni method to account for multiple testing. To evaluate whether the between-group comparisons were influenced by extreme values, a sensitivity analysis was performed in which outliers, defined as values lying outside Q1 − 1.5×IQR to Q3 + 1.5×IQR within each group, were excluded, and the Kruskal–Wallis tests with Bonferroni-adjusted pairwise comparisons were repeated. For correlation analysis, Pearson correlation or Spearman rank correlation methods were employed. Correlation analyses were conducted as exploratory analyses; therefore, no additional correction for multiple comparisons was applied. All statistical tests were two-sided, with a significance level set at p < 0.05, and confidence intervals reported correspond to the 95% level. Given the exploratory nature of this pilot study, a *post hoc* power analysis was performed using G*Power version 3.1.9.7. Based on the observed effect size (Cohen’s d = 0.33) for retinal arterial diameter between GH and PE groups, a total sample size of 292 participants (146 per group) would be required to achieve 80% power at a two-sided alpha level of 0.05.

Prior to the construction of the multivariable models, we evaluated the potential multicollinearity among the independent variables (serum uric acid, diastolic blood pressure, body mass index (BMI), and gestational age) by utilizing the variance inflation factor (VIF) and tolerance. VIF values were calculated by regressing each independent variable on the remaining covariates. All VIF values were below 1.5 (range: 1.116–1.472), and the tolerance values ranged from 0.678 to 0.898, which indicated the absence of significant multicollinearity. Consequently, all four variables were retained for the subsequent analyses. To account for inter-eye correlation and to evaluate the independent associations between clinical parameters and retinal vascular measurements, multivariable generalized estimating equation (GEE) models were constructed. Subject ID was specified as the clustering variable, and an exchangeable working correlation structure was applied. Robust (sandwich) standard errors were used to ensure valid inference under potential misspecification of the correlation structure. Separate GEE models were fitted with the following dependent variables: Retinal arterial diameter (μm); Mean venous diameter within 3 mm of the fovea centralis (μm); Arteriovenous ratio (AVR); Mean arterial branching angle (°); Fractal dimension (FD) in the inferior optic disc region. Serum uric acid (μmol/L), diastolic blood pressure (mmHg), body mass index (BMI, kg/m^2^), and gestational age (weeks) were simultaneously entered as independent variables in all models. Regression coefficients (B), 95% confidence intervals (CI), and two-sided p-values were reported. Statistical significance was defined as p < 0.05.

## 3 Results

### 3.1 Demographic and baseline characteristics

A total of 36 pregnant women were enrolled in this study, comprising 11 individuals (22 eyes) in the healthy control group, 15 individuals (30 eyes) in the GH group, and 10 individuals (20 eyes) in the PE group. The demographic and clinical characteristics of the full cohort are summarized in Table 1. No statistically significant between-group differences were observed for age, height, pre-pregnancy weight, BMI, gestational age, gravidity, parity, and fasting blood glucose levels among the three groups (all *p* > 0.05), indicating a good comparability of baseline data across the groups.

**TABLE 1:** Sociodemographic and clinical characteristics of the study cohort.

Characteristics		Normotensive pregnant control group	Gestational hypertension group	Preeclampsia group	*P* value
Age (years)		27.25 ± 2.71	29.86 ± 4.44	27.43 ± 3.60	0.231
Height (cm)		158.25 ± 5.39	159.36 ± 4.07	158.57 ± 6.05	0.885
Body weight (kg)		72.13 ± 7.85	77.75 ± 8.55	68.44 ± 10.97	0.084
Body mass index (BMI, kg/m^2^)		28.13 ± 4.08	30.67 ± 3.67	27.38 ± 5.02	0.215
Systolic blood pressure (mmHg)		114.38 ± 8.48	132.93 ± 14.89	135.57 ± 18.15	0.008
Diastolic blood pressure (mmHg)		71.38 ± 6.57	92.36 ± 10.05	90.71 ± 12.50	<0.001
Gestational age (weeks)		37.21 ± 2.05	37.39 ± 2.41	37.36 ± 1.49	0.975
Gravidity		1.70 ± 0.82	2.13 ± 1.46	2.10 ± 0.99	0.8
Parity		0.40 ± 0.52	0.47 ± 0.64	0.40 ± 0.52	0.96
Fasting blood glucose (mmol/L)		4.73 ± 0.50	4.83 ± 0.54	4.57 ± 0.43	0.473
Uric acid (μmol/L)		232.73 ± 26.39	335.16 ± 69.96	359.27 ± 119.52	0.0016
Urine protein grade, n (%)	Grade 0	11 (100.0%)	13 (86.7%)	2 (20.0%)	<0.001
	Grade 1	0 (0.0%)	2 (13.3%)	5 (50.0%)	
	Grade 2	0 (0.0%)	0 (0.0%)	2 (20.0%)	
	Grade 3	0 (0.0%)	0 (0.0%)	1 (10.0%)	
24-h urinary protein excretion (mg/24 h)		113.55 ± 36.21	184.14 ± 159.25	2064.48 ± 1859.39	<0.001

Categorical variables are expressed as n (%), continuous variables are expressed as mean ± standard deviation.

As expected, significant between-group differences were observed for blood pressure indices: both systolic blood pressure (SBP) and diastolic blood pressure (DBP) were significantly higher in the GH and PE groups than those in the healthy control group (SBP: *p* = 0.008; DBP: *p* < 0.001). Serum uric acid levels also differed significantly across groups, with 43.9% and 54.4% higher levels observed in the GH and PE groups respectively compared to controls (*p* = 0.002). Urine protein grade distribution showed marked between-group heterogeneity (*p* < 0.001), with 24-h urinary protein excretion in the PE group 11.2-fold higher than in the GH group and 18.2-fold higher than in controls (*p* < 0.001), consistent with more severe renal endothelial injury in PE patients.

### 3.2 Automatic segmentation and quantitative assessment of HDP-Associated retinopathy

**FIGURE 3:**
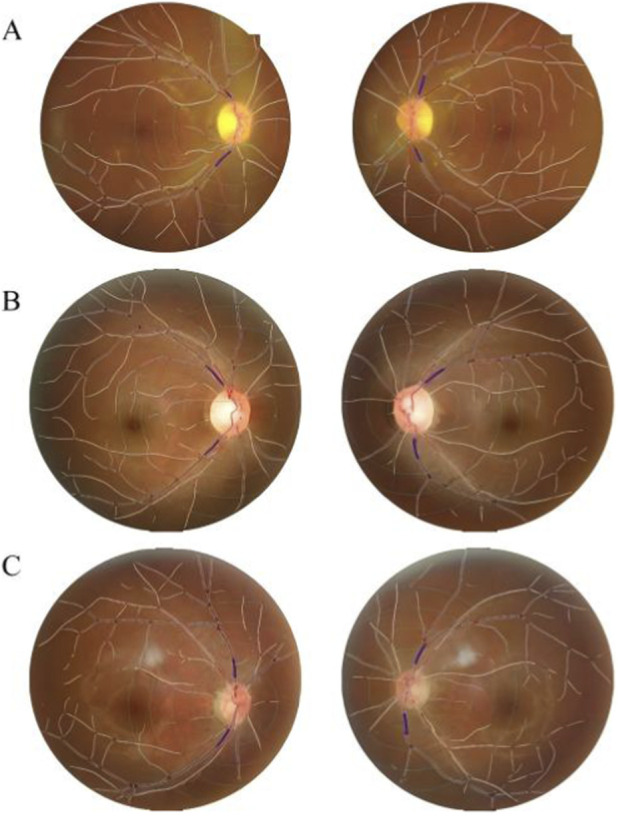
Fundus images of both eyes and quantitative vascular results for representative participants across the three groups. **(A)** Healthy control group; **(B)** Gestational hypertension group; **(C)** Preeclampsia group. One representative participant was selected from each group to display standardized fundus images of the left and right eyes. Retinal arterial vessels are marked in red, and retinal venous vessels are marked in blue. Microvascular parameters including vascular caliber, arteriovenous ratio (AVR) and other indices were quantitatively measured based on this segmentation result.

To assess HDP-related retinal structural alterations, this study conducted a high-dimensional quantitative analysis of fundus images utilizing the automatic segmentation algorithm from EVisionAI (Yiwei Technology (Beijing) Co., Ltd.). More than 300 vascular structural parameters were extracted in total. The results of multi-dimensional comparisons indicated significant differences in various vascular structural indices among different groups. Notably, arterial caliber, average venous caliber within 3 mm of the fovea centralis, and the arteriovenous ratio (AVR) exhibited clear discriminatory trends (Figure 3).

Compared to the healthy control group, both the GH group and the PE group demonstrated decreased arterial and venous calibers, alongside a declining trend in AVR, with the lowest AVR recorded in the GH group (Table 2). In summary, the retinal manifestations of HDP primarily consist of arteriolar narrowing and reduced AVR, suggesting that HDP adversely impacts retinal vascular structure through vasoconstriction and alterations in microcirculation.

**TABLE 2:** Retinal vascular measurements of the representative participants presented in Figure 2 (one participant per group).

Ocular fundus parameters	Normotensive pregnant control		Gestational hypertension group		Preeclampsia group	
	OS	OD	OS	OD	OS	OD
AD (μm)	73.168	75.098	53.561	42.64	60.918	52.043
MVD (μm)	64.379	67.703	50.433	45.102	52.04	54.045
AVR	0.874	0.857	0.709	0.579	0.769	0.646

AD, arterial diameter; MVD, mean venous diameter within 3 mm of the fovea centralis; AVR, arteriovenous diameter ratio. Values represent the measurements of a single representative participant from each group (corresponding to Figure 2, OS, left eye; OD, right eye), shown to illustrate the output of the automated segmentation and quantification pipeline. These are individual measurements, not group-level statistics.

### 3.3 Between-group comparisons

Kruskal–Wallis test results demonstrated that between groups differences were predominantly concentrated in vascular caliber indices (Table 3). Compared to the healthy control group, the GH group exhibited significant narrowing across multiple vascular caliber parameters, including global arterial caliber, average arterial caliber within the 0.5–1.0 PD region, and average venous caliber within 3 mm of the fovea centralis (all *p* < 0.001). This was accompanied by a significant decrease in the arteriovenous ratio (AVR, *p* = 0.001). In addition to caliber alterations, a decrease in FD in the inferior optic disc region, changes in arterial tortuosity (VT), and a reduction in regional vascular density (VD) also reached statistical significance (all *p* < 0.05). These findings suggest that microcirculatory structural alterations, primarily characterized by the narrowing of small vessel caliber, have already developed at the gestational hypertension stage, suggesting that vasoconstriction may represent a structural feature of early-stage disease.

**TABLE 3:** Comparison of fundus parameters among the healthy control group, gestational hypertension group, and preeclampsia group.

Ocular fundus parameters	GH vs. Normotensive pregnant control	PE vs. Normotensive pregnant control	PE vs. GH
FD	0.024*	0.036*	>0.99
AT_0_._5_–_1_._0_PD	0.024*	0.036*	>0.99
VT	0.042*	0.940	0.573
VCDR	0.010*	0.172	>0.99
ABA	>0.99	0.282	0.034*
VD_1_._0_–_1_._5_PD	0.036*	0.346	>0.99
VD_fovea	0.011*	0.401	0.667
AD (μm)	<0.001*	0.004*	>0.99
AD_0_._5_–_1_._0_PD (μm)	<0.001*	0.016*	0.148
MVD (μm)	<0.001*	<0.001*	>0.99
AVR	0.001*	0.113	0.708
FTD	0.055	0.009*	>0.99

FD, vascular fractal dimension in the inferior optic disc region; AT_0_._5_–_1_._0_PD, mean arterial tortuosity at 0.5–1.0 papillary diameter (PD) from the optic disc margin; VT, venous tortuosity; VCDR, vertical cup-to-disc ratio; ABA, mean arterial branching angle; VD_1_._0_–_1_._5_PD, vascular density at 1.0–1.5 PD from the optic disc margin; VD_fovea, vascular density within 5 mm of the fovea centralis; AD, arterial diameter; AD_0_._5_–_1_._0_PD, mean arterial diameter at 0.5–1.0 PD from the optic disc margin; MVD, mean venous diameter within 3 mm of the fovea centralis; AVR, arteriovenous diameter ratio; FTD, density of fundus tessellation. Data are presented as adjusted *P*-values. Between-group comparisons were performed using the Kruskal-Wallis test, followed by Bonferroni-corrected multiple pairwise comparisons.

* indicates statistically significant difference (*p* < 0.05).

**FIGURE 4:**
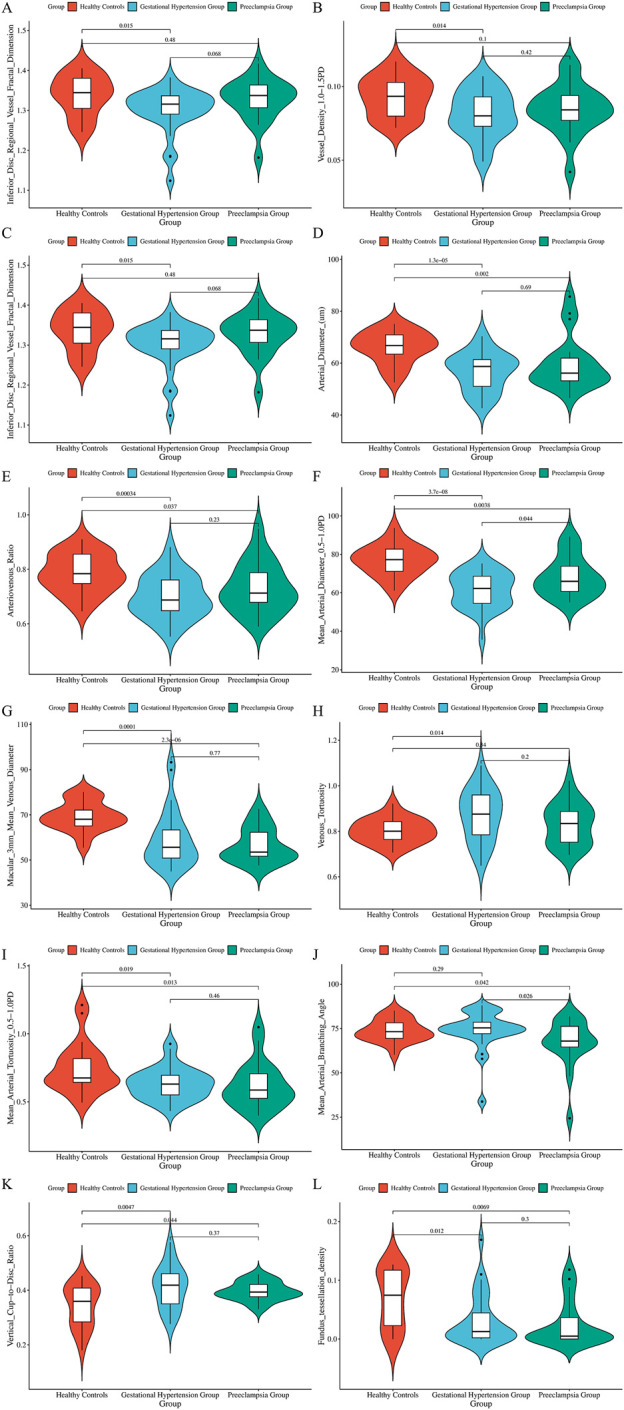
Comparison of retinal microvascular quantitative parameters among Healthy Controls, Gestational Hypertension Group, and Preeclampsia Group. **(A)** Vascular fractal dimension in the inferior optic disc region; **(B)** Vascular density within 1.0–1.5 PD region; **(C)** Vascular density within 5 mm of the fovea centralis; **(D)** Arterial caliber (μm); **(E)** Arteriovenous ratio (AVR); **(F)** Average arterial caliber within 0.5–1.0 PD region; **(G)** Average venous caliber within 3 mm of the fovea centralis; **(H)** Venous tortuosity; **(I)** Average arterial tortuosity within 0.5–1.0 PD region; **(J)** Average arterial branching angle; **(K)** Vertical cup-to-disc ratio; **(L)** Fundus tessellation density. Horizontal lines indicate pairwise between-group comparisons, with Bonferroni-adjusted P-values labeled. The violin plot with embedded box plot displays the interquartile range, and the median is indicated by the central horizontal line.

Compared to the healthy control group, the PE group exhibited significantly reduced arterial caliber, average arterial caliber within the 0.5–1.0 PD region, and average venous caliber within 3 mm of the fovea centralis (all *p* < 0.05). Additionally, a decrease in FD in the inferior optic disc region was observed, indicating that vascular caliber narrowing is a stable structural phenotype during the progression of HDP. Further comparison between the GH group and the PE group revealed that the differences in most caliber indices and the arteriovenous ratio (AVR) between the two groups did not reach statistical significance (all *p* > 0.05), suggesting a high degree of consistency in their overall vasoconstriction patterns. Moreover, only the average arterial branching angle demonstrated a significant difference between the two groups (*p* = 0.034), indicating that, alongside alterations in vascular caliber, remodeling of vascular geometric configuration contributes to the structural stratification associated with disease severity. Violin plots further validated these results at the distribution level: arterial caliber and macular venous caliber exhibited clear separation between the healthy control group and both types of HDP patients, while substantial overlap was noted between the two disease groups; AVR displayed an overall downward shift in the disease groups (Figure 4).

In summary, the narrowing of retinal vascular caliber represents a consistent structural alteration observed across HDP groups in this cross-sectional cohort. Changes in vascular geometric parameters, such as the average arterial branching angle, reflect structural remodeling features during disease progression and serve as important biomarkers for assessing disease progression. Notably, global arterial caliber, average arterial caliber within the 0.5–1.0 PD region, average venous caliber within 3 mm of the fovea centralis, arterial-to-venous ratio (AVR), and FD in the inferior optic disc region may serve as candidate imaging markers for further validation in prospective studies. Furthermore, the mean arterial branching angle may reflect structural differences between GH and PE that merit further investigation.

A sensitivity analysis that excluded outliers (using the 1.5×IQR rule) produced results that were consistent with those of the primary analysis for the vast majority of parameters. Specifically, the mean arterial branching angle continued to show a significant difference between the GH and PE groups (Bonferroni-adjusted *p* = 0.023), and the narrowing of arterial and venous calibers remained highly significant (all *p* < 0.001). The sole exception was the vascular fractal dimension in the inferior optic disc region. After the exclusion of outliers, the difference between groups was diminished and no longer achieved statistical significance (*p* = 0.081), which implies that the association of the inferior optic disc fractal dimension with group status might be less stable and potentially affected by distributional extremes.

### 3.4 Correlation analysis

Spearman correlation analysis revealed that quantitative retinal vascular parameters exhibited significant correlation patterns with clinical indicators, which were highly consistent with the results of between-group comparisons (Table 4). For caliber-related parameters, arterial caliber, average arterial caliber within the 0.5–1.0 PD region, and average venous caliber within 3 mm of the fovea centralis all demonstrated significant negative correlations with diastolic blood pressure (*r* = −0.436, −0.441, −0.535, respectively; all p < 0.001), as well as significant negative correlations with systolic blood pressure. Notably, the correlation between serum uric acid and the aforementioned caliber indices was consistently stronger than that observed with blood pressure: the correlation coefficient between arterial caliber and uric acid was the highest (*r* = −0.614, p < 0.001), while the correlations of average arterial caliber within 0.5–1.0 PD (*r* = −0.478) and macular venous caliber (*r* = −0.458) with uric acid were all stronger than or comparable to the corresponding correlation coefficients with blood pressure. The arteriovenous ratio also exhibited significant negative correlations with systolic blood pressure (*r* = −0.265), diastolic blood pressure (*r* = −0.324), and uric acid (*r* = −0.444). These results suggest that in the HDP population, serum uric acid showed stronger univariate correlations with retinal caliber parameters compared with blood pressure.

**TABLE 4:** Correlation analysis between fundus parameters and sociodemographic and clinical characteristics.

Ocular fundus parameters	BMI		GA		SBP		DBP		UA		24hUP	
	*r*	*p*	*r*	*p*	*r*	*p*	*r*	*p*	*r*	*p*	*r*	*p*
FD	−0.025	0.833	−0.075	0.533	−0.048	0.686	−0.036	0.765	−0.087	0.516	−0.077	0.613
VD_1_._0_–_1_._5_pd	−0.061	0.609	−0.107	0.371	−0.187	0.116	−0.167	0.162	−0.154	0.247	−0.395**	0.007
VD_fovea	−0.232	0.051	−0.185	0.123	−0.222	0.062	−0.197	0.099	−0.181	0.179	−0.320*	0.032
AD	−0.169	0.157	−0.378**	0.001	−0.236*	0.046	−0.436**	<0.001	−0.614**	<0.001	−0.303*	0.041
AVR	−0.15	0.21	−0.343**	0.003	−0.265*	0.024	−0.324**	0.005	−0.444**	<0.001	−0.315*	0.033
AD_0_._5_–_1_._0_pd	−0.115	0.337	−0.367**	0.002	−0.225	0.057	−0.441**	<0.001	−0.478**	<0.001	−0.237	0.112
MVD	−0.188	0.117	−0.171	0.154	−0.273*	0.021	−0.535**	<0.001	−0.458**	<0.001	−0.476**	0.001
VT	0.011	0.925	0.208	0.08	0.08	0.506	0.386**	0.001	0.236	0.074	−0.046	0.762
AT_0_._5_–_1_._0_pd	−0.158	0.186	−0.1	0.405	−0.221	0.062	−0.247*	0.036	−0.307*	0.019	−0.410**	0.005
ABA	0.146	0.226	0.048	0.693	−0.173	0.153	−0.141	0.245	−0.087	0.521	−0.389**	0.007
VCDR	0.03	0.803	0.423**	<0.001	0.262*	0.026	0.404**	<0.001	0.095	0.479	0.078	0.605
FTD	−0.046	0.7	−0.228	0.054	−0.275*	0.02	−0.296*	0.012	−0.02	0.882	−0.234	0.118

FD, vascular fractal dimension in the inferior optic disc region; VD_1_._0_–_1_._5_PD, vascular density at 1.0–1.5 PD, from the optic disc margin; VD_fovea, vascular density within 5 mm of the fovea centralis; AD, arterial diameter; AVR, arteriovenous diameter ratio; AD_0_._5_–_1_._0_PD, mean arterial diameter at 0.5–1.0 PD, from the optic disc margin; MVD, mean venous diameter within 3 mm of the fovea centralis; VT, venous tortuosity; AT_0_._5_–_1_._0_PD, mean arterial tortuosity at 0.5–1.0 PD, from the optic disc margin; ABA, mean arterial branching angle; VCDR, vertical cup-to-disc ratio; FTD, density of fundus tessellation; GA, gestational age; SBP, systolic blood pressure; DBP, diastolic blood pressure; UA, serum uric acid; 24hUP, 24-h urinary protein. *R* indicates the correlation coefficient, *P* indicates the result of the significance test. Spearman correlation analysis was used in this study.

* indicates *p* < 0.05.

** indicates *p* < 0.01.

A distinct correlation pattern was observed for vascular density and geometric structure parameters, differing from caliber-related parameters. Vascular density within the 1.0–1.5 PD region (*r* = −0.395, *p* = 0.007), vascular density within 5 mm of the fovea centralis (*r* = −0.320, *p* = 0.032), and the average arterial branching angle (*r* = −0.389, *p* = 0.007) all exhibited significant negative correlations with 24-h urinary protein excretion, while no significant associations were found with blood pressure or uric acid levels. Conversely, the average venous caliber within 3 mm of the fovea centralis demonstrated significant correlations with diastolic blood pressure (*r* = −0.535), uric acid (*r* = −0.458), and 24-h urinary protein (*r* = −0.476) simultaneously, indicating that macular venous caliber may reflect both abnormal vascular tone and systemic endothelial injury. Additionally, average arterial tortuosity within the 0.5–1.0 PD region showed significant negative correlations with diastolic blood pressure (*r* = −0.247), uric acid (*r* = −0.307), and 24-h urinary protein (*r* = −0.410), suggesting that altered arterial tortuosity may represent a structural manifestation of the combined effects of multiple pathological mechanisms. Furthermore, the vertical cup-to-disc ratio exhibited significant positive correlations with diastolic blood pressure (*r* = 0.404, *p* < 0.001), systolic blood pressure (*r* = 0.262, *p* = 0.026), and gestational age (*r* = 0.423, *p* < 0.001), displaying an opposite direction of association compared to other parameters. This suggests that it may reflect changes related to optic disc perfusion or intraocular pressure independent of the vasoconstriction mechanism.

In summary, a distinct dual-track correlation pattern exists between quantitative retinal parameters and clinical indicators. Caliber-related parameters predominantly correlate with blood pressure and uric acid levels, indicating abnormalities in vascular tone regulation. Conversely, vascular density and branching geometric parameters primarily correlate with proteinuria, suggesting a systemic endothelial injury burden. This dissociation pattern implies that various types of fundus parameters represent two parallel pathological dimensions of HDP, providing complementary clinical assessment value.

### 3.5 Multivariable GEE analysis

Multivariable GEE analyses were performed to evaluate independent associations while accounting for inter-eye correlation (Table 5). Serum uric acid remained independently associated with narrower retinal arterial caliber (B = −0.034 μm per μmol/L increase, 95% CI: −0.067 to −0.001, *p* = 0.042), whereas diastolic blood pressure, BMI, and gestational age were not significant in this model. In contrast, diastolic blood pressure was independently associated with reduced mean venous diameter within 3 mm of the fovea centralis (B = −0.383 μm per mmHg increase, 95% CI: −0.634 to −0.133, *p* = 0.003), while serum uric acid was not significant. No independent associations were observed for AVR or fractal dimension. Diastolic blood pressure showed a borderline association with mean arterial branching angle (*p* = 0.051). These results suggest differential association patterns between arterial and venous parameters in multivariable analysis.

**TABLE 5:** Generalized Estimating Equations (GEE) analysis of associations between fundus parameters and clinical variables.

Dependent variable	Independent variable	B (95% CI)	*P* value
AD (μm)	UA (per μmol/L)	−0.034 (−0.067, −0.001)	0.042
	DBP (per mmHg)	−0.062 (−0.371, 0.247)	0.695
	BMI (kg/m^2^)	−0.088 (−0.674, 0.498)	0.769
	GA (weeks)	−0.537 (−1.109, 0.036)	0.066
MVD (μm)	UA (per μmol/L)	−0.017 (−0.045, 0.011)	0.230
	DBP (per mmHg)	−0.383 (−0.634, −0.133)	0.003
	BMI (kg/m^2^)	−0.048 (−0.459, 0.364)	0.821
	GA (weeks)	−0.221 (−0.695, 0.254)	0.362
AVR	UA (per μmol/L)	−0.0001 (−0.0003, 0.00002)	0.073
	DBP (per mmHg)	−0.0003 (−0.003, 0.002)	0.851
	BMI (kg/m^2^)	0.00001 (−0.002, 0.002)	0.991
	GA (weeks)	−0.005 (−0.011, 0.001)	0.129
ABA (°)	UA (per μmol/L)	0.039 (−0.017, 0.096)	0.172
	DBP (per mmHg)	−0.395 (−0.792, 0.002)	0.051
	BMI (kg/m^2^)	0.168 (−0.363, 0.699)	0.535
	GA (weeks)	−0.151 (−0.568, 0.266)	0.477
FD	UA (per μmol/L)	−0.00002 (−0.001, 0.001)	0.974
	DBP (per mmHg)	−0.0001 (−0.001, 0.001)	0.834
	BMI (kg/m^2^)	0.00001 (−0.001, 0.001)	0.956
	GA (weeks)	−0.002 (−0.007, 0.002)	0.312

AD, arterial diameter; MVD, mean venous diameter within 3 mm of the fovea centralis; AVR, arteriovenous diameter ratio; ABA, mean arterial branching angle; FD, fractal dimension in the inferior optic disc region; UA, serum uric acid; DBP, diastolic blood pressure; BMI, body mass index; GA, gestational age. Multivariable generalized estimating equation (GEE) models were constructed with subject ID, as the clustering variable using an exchangeable working correlation structure and robust standard errors. All listed independent variables were entered simultaneously into each model. B indicates unstandardized regression coefficient; CI, confidence interval.

### 3.6 Sensitivity analyses for inter-eye dependence

To evaluate the robustness of our findings and tackle potential inter - eye dependence, we carried out two supplementary sensitivity analyses: one employing a randomly chosen eye for each participant and the other utilizing bilateral mean values. Across both methods, arterial diameter, the mean arterial diameter within the 0.5–1.0 PD region, the mean venous diameter within 3 mm of the fovea centralis, and the arteriovenous ratio (AVR) continued to exhibit significant differences among groups. These results were consistent not only with the primary GEE models but also with the initial inter - group comparisons conducted using bilateral eye data. Correlation analyses in both sensitivity models further verified that retinal arterial caliber remained negatively correlated with serum uric acid and diastolic blood pressure, while venous caliber remained correlated with diastolic blood pressure and 24 - hour urinary protein excretion. These patterns were in line with the correlation analyses based on bilateral eye data. Collectively, the consistency of results across bilateral - eye analyses, GEE modeling, randomly selected single - eye analyses, and bilateral mean - value approaches validates the robustness of the main findings of this study, specifically, the consistent narrowing of retinal vascular caliber in HDP and its differential metabolic and hemodynamic associations.

## 4 Discussion

HDP are characterized by systemic endothelial dysfunction and microvascular abnormalities, which are core pathological features. Microcirculatory damage is present throughout the entire process of disease onset and progression (Yu et al., 2018). Retinal vessels exhibit anatomical and physiological similarities to cerebral vessels and can be directly observed using non-invasive methods, making them widely recognized as a natural window for reflecting systemic microcirculatory status (Yuan et al., 2024). However, previous studies have predominantly relied on manual grading methods, which can only detect qualitative alterations, such as retinal arteriolar spasm and hypoperfusion, in patients with preeclampsia. Furthermore, studies focusing on automated quantitative analysis with continuous variables across different stages of HDP remain limited (Roberts and Hubel, 2009; Redman and Sargent, 2005). This study employed artificial intelligence-assisted automatic segmentation technology to conduct a standardized quantitative assessment of retinal microvascular parameters across various stages of HDP. This methodological framework aligns with the recent expert consensus that advocates for the standardized validation and responsible clinical integration of artificial intelligence systems in ophthalmology (Shao et al., 2025). This approach reveals their structural characteristics at the continuous variable level, providing objective quantitative evidence for understanding microcirculatory alterations related to HDP.

Retinal vascular caliber narrowing as a consistent microvascular phenotype associated with HDP. The present study found that arterial caliber, average arterial caliber within the 0.5–1.0 PD region, and average venous caliber within 3 mm of the fovea centralis were significantly lower in the GH group compared to the healthy control group (all *p* < 0.001). These alterations persisted into the PE stage. However, the differences in these indices between the GH and PE groups did not reach statistical significance (all *p* > 0.05). This pattern suggests that retinal vascular caliber narrowing is the core microcirculatory phenotype of HDP that is observed across different HDP stages in this cross-sectional cohort, rather than serving as a dose-response indicator that progressively worsens with increasing disease severity. This finding supports the HDP continuum theory at the imaging level, indicating that GH and PE may share a common microvascular constriction basis, with PE representing an amplification of inflammation and increased severity of target organ involvement, rather than an independent vascular structural pattern (Benschop et al., 2017). Notably, there were no statistically significant differences in baseline data (age, BMI, gestational age, fasting blood glucose, etc.) among the three groups, thereby ruling out the influence of major confounding factors on these conclusions and enhancing the robustness of the between-group comparison results.

Serum uric acid showed stronger univariate correlations with retinal arterial caliber compared with blood pressure. Correlation analysis revealed that the relationship between serum uric acid and arterial caliber (*r* = −0.614, *p* < 0.001) was the strongest among all clinical indicators, surpassing that of systolic blood pressure (*r* = −0.236) and diastolic blood pressure (*r* = −0.436). For the average arterial caliber within the 0.5–1.0 PD region (uric acid *r* = −0.478 vs. diastolic blood pressure *r* = −0.441) and the arteriovenous ratio (AVR) (uric acid *r* = −0.444 vs. diastolic blood pressure *r* = −0.324), the correlation with uric acid was also stronger than that with blood pressure indices. The correlation between macular venous caliber and diastolic blood pressure (*r* = −0.535) was similar to that with uric acid (*r* = −0.458), suggesting that venous caliber may be regulated by both blood pressure and metabolic factors. Consistent with these findings, our baseline data indicated that uric acid levels in the GH group and the PE group were significantly higher than those in the healthy control group (335.16 ± 69.96 μmol/L, 359.27 ± 119.52 μmol/L vs. 232.73 ± 26.39 μmol/L, *p* = 0.002), with no statistically significant difference in uric acid levels between the two disease groups, aligning closely with the inter-group distribution pattern of vascular caliber indices. Mechanistically, elevated uric acid can impair vascular endothelial function through mechanisms such as inhibiting nitric oxide (NO) synthesis, activating oxidative stress pathways, and promoting endothelial inflammatory responses, which subsequently leads to persistent arteriolar constriction (Bahadoran et al., 2022). Previous studies have confirmed that hyperuricemia is independently associated with an increased risk of preeclampsia, although direct evidence at the retinal microvascular imaging level remains limited (Colmenares-Mejia et al., 2023). The findings of this study indicate that elevated uric acid levels may be associated with retinal microvascular alterations for retinal microvascular constriction associated with HDP. Furthermore, the combined application of quantitative fundus assessment and uric acid monitoring may provide a potential direction for future prospective investigation. Importantly, in multivariable generalized estimating equation (GEE) models accounting for inter-eye correlation and maternal covariates, serum uric acid remained independently associated with retinal arterial caliber, whereas diastolic blood pressure was independently associated with macular venous caliber. No independent associations were observed for arteriovenous ratio or fractal dimension. These findings refine the univariate correlation results by suggesting differential arterial–venous association patterns after adjustment, indicating that metabolic and hemodynamic factors may relate to distinct retinal vascular components in hypertensive disorders of pregnancy (HDP).

Vascular density and geometric parameters reflect the systemic endothelial injury load and are independently associated with the severity of proteinuria. Unlike caliber-related parameters, vascular density and geometric structure parameters exhibit a distinct correlation pattern. Vascular density within the 1.0–1.5 PD region (*r* = −0.395, *p* = 0.007), vascular density within 5 mm of the fovea centralis (*r* = −0.320, *p* = 0.032), and the average arterial branching angle (*r* = −0.389, *p* = 0.007) were all significantly negatively correlated with 24-h urinary protein excretion, showing no significant association with blood pressure or uric acid levels. These results suggest that reduced retinal vascular density and altered branching geometric configuration reflect the overall load of systemic endothelial injury, rather than isolated changes in vascular tone alone. In this study, the 24-h urinary protein level in the PE group was significantly higher than that in the other two groups (2064.48 ± 1859.39 mg/24 h, *p* < 0.001), indicating a potential cross-organ parallel injury pattern between the degree of renal endothelial involvement and structural alterations in the retinal microvascular network. Additionally, the average arterial branching angle was the only parameter that showed a significant difference between the GH and PE groups in this study (*p* = 0.034), suggesting that remodeling of the vascular geometric configuration may contribute to structural stratification related to disease severity. As a geometric indicator reflecting adaptive remodeling of the vascular network, the branching angle may represent a structural difference between GH and PE that requires validation in larger prospective cohorts.

The arteriovenous ratio (AVR) has long been recognized as a classic biomarker for hypertensive vascular remodeling (Parr and Spears, 1974; Hubbard et al., 1999; Wong et al., 2005). However, this study revealed that AVR exhibited a statistically significant difference only between the GH group and the healthy control group (*p* = 0.001), while it did not reach statistical significance in the PE group (*p* = 0.113). Coupled with the observation that both arterial and venous calibers were simultaneously narrowed in the PE group, these results imply that synchronous arteriovenous constriction may diminish the discriminatory capacity of proportional indicators: when both vessels narrow proportionately, the AVR tends to remain stable, thereby obscuring the true extent of vasoconstriction. In contrast, absolute caliber parameters demonstrated stable significant differences in both GH and PE stages, exhibiting stronger correlations with clinical indicators such as blood pressure and uric acid. These findings suggest that in populations with pregnancy-specific hypertension, absolute vascular caliber may be more sensitive and robust than AVR, thereby providing new empirical evidence for prior discussions regarding the limitations of AVR (Ikram et al., 2013).

Although previous studies have confirmed that women with HDP exhibit retinal microvascular alterations associated with an elevated long-term cardiovascular risk, existing evidence primarily relies on manual grading or single-parameter assessments and lacks a systematic quantitative evaluation of microvascular phenotypes across different stages of HDP (Bellamy et al., 2007; Brown et al., 2013). Utilizing artificial intelligence-powered automatic segmentation and standardized quantification pipelines, this study systematically identifies the distinct clinical significance of three categories of parameters—caliber, density, and geometric configuration—at the continuous variable level, suggesting that serum uric acid may be independently associated with retinal arterial caliber in multivariable analysis (Poplin et al., 2018; Rim et al., 2020; Yuan et al., 2025). These parameters are anticipated to play crucial roles in the management of HDP: during pregnancy, they may provide a structural basis for future evaluation of risk stratification strategies in prospective studies; postpartum, they can be utilized as objective indicators for long-term cardiovascular risk follow-up. The combined application of uric acid monitoring and quantitative fundus assessment may further establish a metabolic-imaging multimodal risk assessment strategy. However, it is important to note that, given the cross - sectional nature of this study, temporal precedence and predictive value cannot be determined. Therefore, the present findings should be interpreted as hypothesis - generating associations rather than evidence of causality or clinical predictive utility. The causal relationship between uric acid levels and the narrowing of retinal vascular caliber necessitates verification through longitudinal studies. Furthermore, the absence of data on angiogenic factors (sFlt1/PlGF) and inflammatory markers limits the integrated analysis of molecular imaging. The present study included 30 GH eyes and 20 PE eyes, which was substantially smaller than the sample size estimated by the *a priori* power analysis (146 per group). Therefore, the absence of statistically significant differences between GH and PE for most retinal parameters should be interpreted with caution, as it may reflect limited statistical power rather than true biological equivalence. The relatively small observed effect size (d = 0.33) further suggests that the magnitude of vascular differences between GH and PE may be modest. In addition, information on gestational weight gain, detailed nutritional status, and antihypertensive medication use was not systematically incorporated into the multivariable models, which may represent residual confounding. Future prospective studies with comprehensive longitudinal data collection are warranted to further isolate independent vascular effects. Currently, the longitudinal follow-up of the enrolled cohort at our center is progressing systematically, and we have concurrently initiated a multicenter clinical collaboration. In future studies, we will expand our sample size to include more cases of HDP from various regions with diverse clinical characteristics. We aim to combine prospectively collected molecular biomarker data with follow-up information on pregnancy outcomes to further validate the causal association between retinal microvascular parameters and the onset and progression of HDP. This will enable a comprehensive evaluation of their independent predictive value for early risk warning and disease severity stratification in HDP. Additionally, we will explore the development of a multidimensional integrated prediction model that incorporates clinical characteristics, molecular biomarkers, and retinal imaging features, thereby providing reliable tools for clinical early warning and precision intervention in HDP.

## 5 Conclusion

In this study, we utilized AI-assisted quantitative retinal microvascular analysis to identify three categories of imaging biomarkers, each with distinct clinical implications across various stages of HDP. Retinal vascular caliber narrowing was consistently observed across HDP groups in this cross-sectional analysis, indicating that GH and PE share a common microvascular pathological basis, thus representing a potential candidate marker pending prospective validation. The arterial branching angle is the sole parameter exhibiting a significant difference between GH and PE, suggesting its potential value for disease progression stratification. Furthermore, the independent correlation between vascular density and proteinuria suggests a potential cross-organ parallel injury pattern. Additionally, Serum uric acid was independently associated with retinal arterial caliber in multivariable models, underscoring the potential application of combined metabolic and imaging assessments in the early risk stratification of HDP. The consistency of results across generalized estimating equation models, randomly selected single-eye analyses, and bilateral mean - value sensitivity analyses further strengthens the robustness of the present findings. This study identifies candidate imaging markers associated with HDP that warrant validation in prospective longitudinal studies. Future longitudinal studies are required to determine whether vascular caliber-related indices could be integrated into routine antenatal care for early identification of high-risk populations, thereby potentially advancing the intervention window to mitigate the risk of adverse maternal and infant outcomes. Meanwhile, the arterial branching angle can be utilized to differentiate disease severity and may offer supplementary structural information pertinent to disease characterization. These findings possess significant practical value for enhancing early warning and precision management of HDP.

## Data Availability

The raw data supporting the conclusions of this article will be made available by the authors, without undue reservation.
